# Enfortumab Vedotin–Induced Toxic Epidermal Necrolysis in Metastatic Urothelial Carcinoma Complicated by Severe Gastrointestinal Bleeding

**DOI:** 10.1002/iju5.70036

**Published:** 2025-05-05

**Authors:** Aika Matsuyama, Takashi Kato, Rion Kawase, Mikinori Kobayashi, Ayako Momota, Yukiko Tsunoda, Asaomi Yamaguchi, Hiroki Hirabayashi, Shoji Suzuki, Masashi Kato

**Affiliations:** ^1^ Department of Urology Japanese Red Cross Aichi Medical Center Nagoya Daiichi Hospital Nagoya Japan; ^2^ Department of Female Urology Japanese Red Cross Aichi Medical Center Nagoya Daiichi Hospital Nagoya Japan

**Keywords:** enfortumab vedotin, gastrointestinal bleeding, toxic epidermal necrolysis, urothelial carcinoma

## Abstract

**Introduction:**

Enfortumab vedotin (EV) has been reported to cause skin toxicity in some patients. We report a rare case of toxic epidermal necrolysis (TEN) induced by EV and complicated by severe gastrointestinal (GI) bleeding.

**Case Presentation:**

A 70‐year‐old man with recurrent urothelial carcinoma developed a trunk rash at 16 days after EV administration. He presented to the emergency department with loss of consciousness and was diagnosed with TEN and septic shock. Although pulse steroid therapy improved his skin lesions, his abdominal symptoms progressively worsened. On Day 27, he developed massive GI bleeding. Despite intensive interventions, he died of multiple organ failure on Day 30.

**Conclusion:**

This case highlights that Stevens–Johnson syndrome/TEN induced by EV can develop shortly after treatment, with delayed and potentially fatal GI manifestations. Given the challenges in managing established TEN, close monitoring for adverse events is essential.

**Summary**

- Significant skin toxicity can develop shortly after enfortumab vedotin administration.
- Careful monitoring of cutaneous manifestations is essential.

**Abbreviations**
EV: enfortumab vedotin
GI: gastrointestinal
IVIG: intravenous immunoglobulin
SJS: Stevens–Johnson syndrome
TEN: toxic epidermal necrolysis

## 1 Introduction

Enfortumab vedotin (EV) is an antibody–drug conjugate approved for the treatment of locally advanced or metastatic urothelial carcinoma. EV causes dermatologic toxicity due to its targeting of nectin‐4. Stevens–Johnson syndrome (SJS) and toxic epidermal necrolysis (TEN) represent a rare but severe spectrum of hypersensitivity reactions characterized by widespread epidermal detachment. TEN often leads to sepsis and multi‐organ failure, resulting in a mortality rate of 20%–40% [1]. Severe gastrointestinal (GI) symptoms occur in approximately 10% of patients with SJS/TEN [2], and can be life‐threatening.

## 2 Case Presentation

A 70‐year‐old man with no known allergy presented with complaints of macrohematuria. The patient was diagnosed with right renal pelvic urothelial carcinoma, staged as cT3N0M0. He underwent four cycles of neoadjuvant chemotherapy with dose‐dense MVAC (methotrexate 30 mg/m^2^, doxorubicin 50 mg/m^2^, vinblastine 3 mg/m^2^, and cisplatin 70 mg/m^2^), followed by laparoscopic total nephroureterectomy. The postoperative diagnosis was invasive urothelial carcinoma, stage ypT3N0M0. The patient received three cycles of adjuvant therapy with nivolumab at a dose of 480 mg every 4 weeks. However, metastasis to the left ilium was detected, prompting initiation of enfortumab vedotin at 1.25 mg/kg.

**FIGURE 1:**
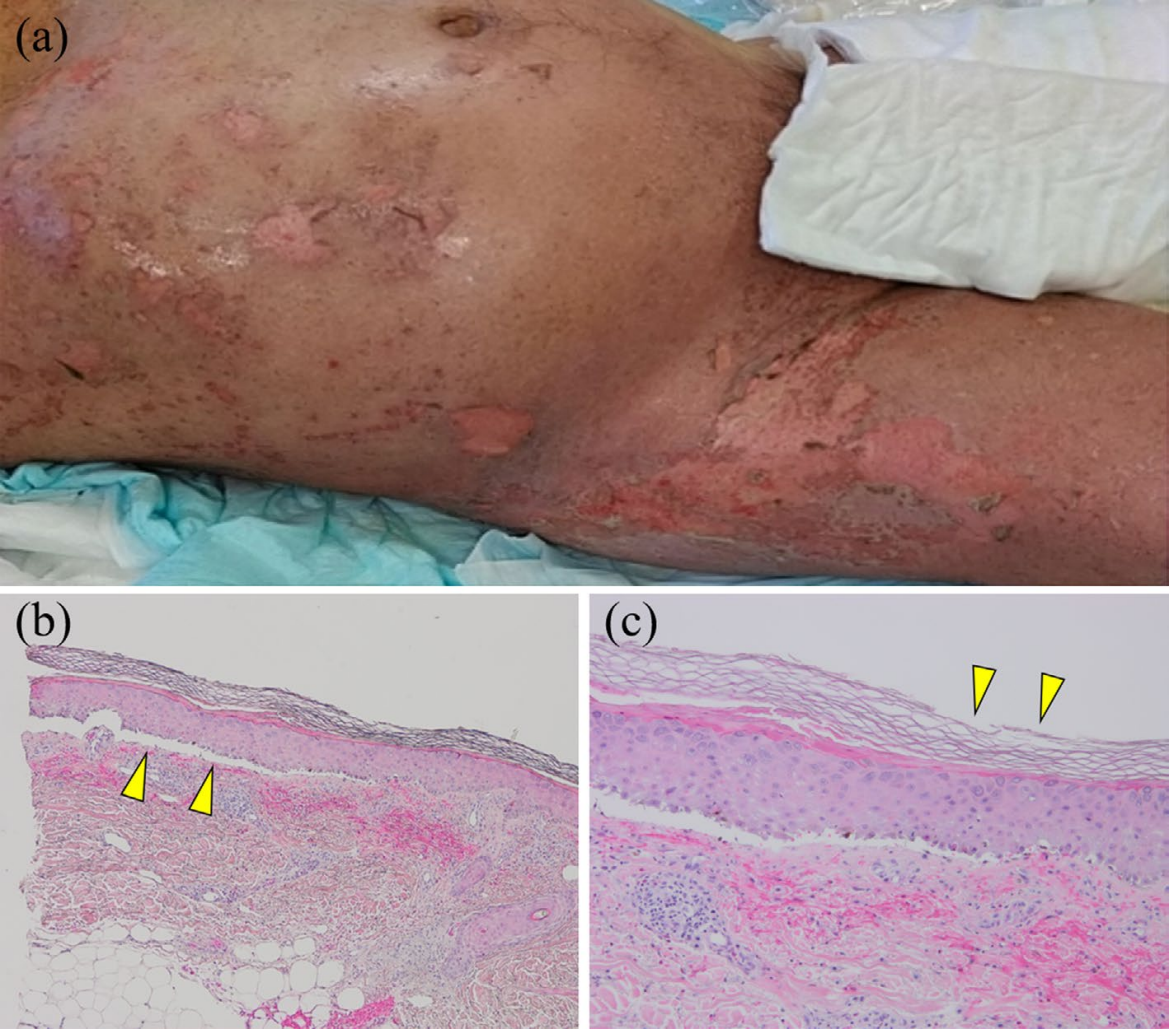
(a) Macroscopic findings of the skin on Day 18 after EV administration. Widespread erythema with epidermolysis and erosion were observed on the trunk and limbs. (b) Microscopy images of the cutaneous biopsy performed on day 18. Cleft formation between the dermis and epidermis (hematoxylin–eosin stain; original magnification ×40). (c) Lymphocytic infiltration into the basement membrane and widespread necrosis of keratinocytes (hematoxylin–eosin stain; original magnification ×100).

**FIGURE 2:**
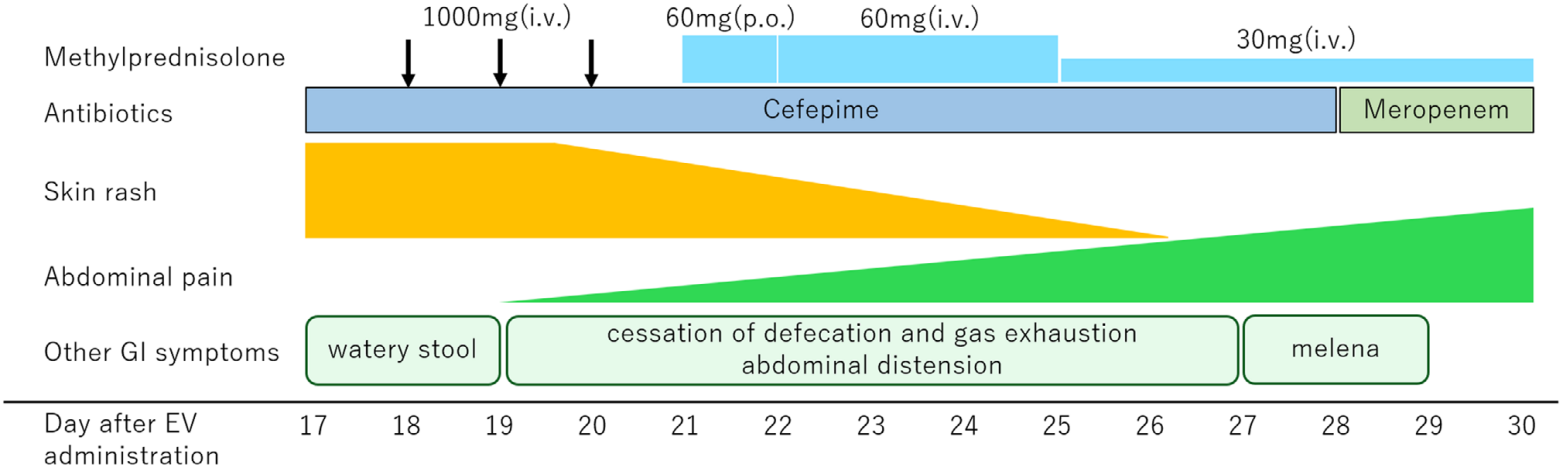
Treatment course of the patient after hospitalization.

On the 16th day following the initial administration of EV, the patient developed a skin rash on his trunk. On the 17th day, he lost consciousness and was transported to the emergency department. Upon arrival, he had a fever of 39.7°C, blood pressure of 68/42 mmHg, a heart rate of 140 bpm, and SpO_2_ of 87% on room air. Physical examination revealed widespread erythema with epidermolysis and erosion on the trunk and limbs, although the mucosa remained unaffected. The skin lesions covered more than 30% of his body surface area. He was hospitalized, and a cutaneous biopsy performed on Day 18 showed cleft formation between the dermis and epidermis, lymphocytic infiltration at the basement membrane, and widespread necrosis of keratinocytes (Figure 1). He was diagnosed with TEN and septic shock, and pulse steroid therapy with methylprednisolone (1000 mg/day) was administered. As the skin rash gradually improved and he was complicated by sepsis and febrile neutropenia, methylprednisolone was tapered to 60 mg/day on Day 21, and further decreased to 30 mg/day on Day 24 (Figure 2).

**FIGURE 3:**
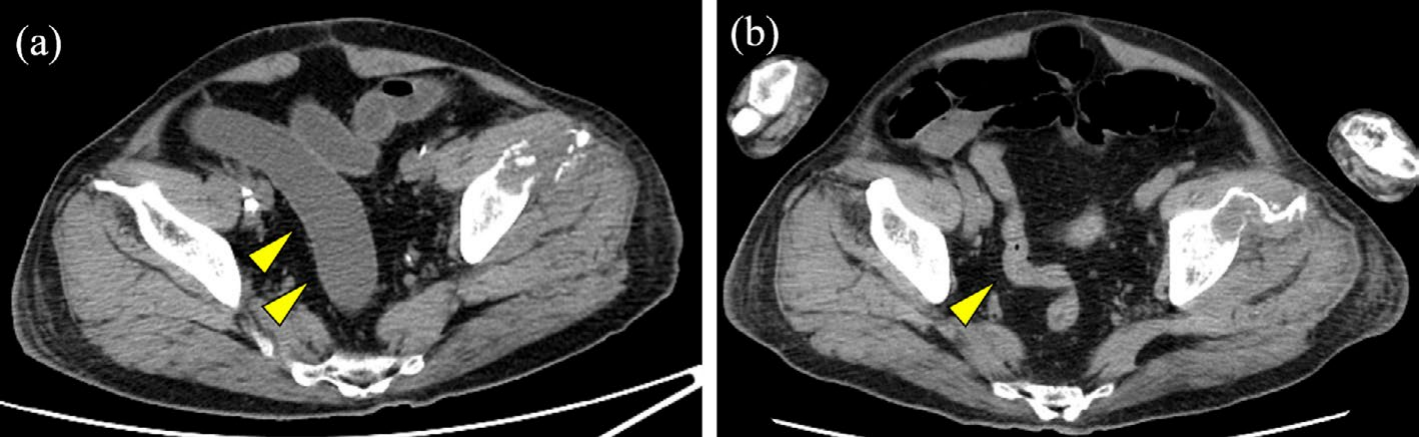
(a) Abdominal CT scan on admission (Day 17) showing edematous intestinal mucosa, dilation, and fluid accumulation inside in the sigmoid colon and rectum. (b) Abdominal CT scan on Day 25 revealing mucosal edema and obstruction extending from the sigmoid colon to the rectum.

While the skin rash was improving, the GI symptoms worsened. Watery stools were noted on admission, and a computed tomography (CT) scan showed edematous intestinal mucosa, dilation, and fluid accumulation inside the sigmoid colon and rectum. By Day 19, defecation and gas exhaustion had ceased, and the patient gradually began experiencing abdominal pain and distension. A subsequent CT scan revealed obstruction extending from the sigmoid colon to the rectum (Figure 3). Intestinal mucosal injury associated with TEN was suspected, and a nasogastric tube was inserted to reduce the intestinal pressure. On Day 27, the patient experienced a significant melena, raising suspicion of bleeding from an ischemic colon. However, colonoscopy was contraindicated because of the high risk of intestinal perforation. The patient's condition rapidly deteriorated, progressing to septic and hemorrhagic shock. Despite intensive interventions—including intubation, plasmapheresis, hemodialysis, and blood transfusion—he died of multiple organ failure on Day 30.

## 3 Discussion

EV is an antibody–drug conjugate that targets nectin‐4‐expressing cells, disrupting cell cycle progression. Nectin‐4 is overexpressed in malignant tumors but is also present in epidermal keratinocytes, which may contribute to skin toxicity [3]. SJS and TEN are severe skin reactions typically induced by drugs, characterized by epidermal necrosis and mucosal erosion. SJS is defined as affecting < 10% of the body surface area, while TEN involves ≥ 30%. The prognosis of TEN is poor, with a reported mortality rate of 20%–40% [1]. Treatment strategies for SJS/TEN involve immediate discontinuation of the suspected drug, suppression of the inflammatory response, and systemic management including systemic steroid therapy, plasmapheresis, and IVIG. Cyclosporine and etanercept are potentially effective, although these treatments have not been fully studied.

Nguyen et al. [4] summarized eight cases of SJS/TEN caused by EV, with a median onset time of 11 days (range: 9–21) from the administration of EV. We identified five additional cases [5, 6, 7, 8] (Table 1). Onset occurred within the first cycle in four patients; the remaining patient developed symptoms at 5 weeks after administration. In our case, the skin rash appeared on Day 16 of the first cycle, aligning with previous reports. Cutaneous adverse events are also observed in patients taking combination therapy with EV and pembrolizumab. In the EV‐302 trial [9], grade ≥ 3 skin reactions occurred in 16.1% of patients (71 of 440), with a median onset time of 1.61 months (range: 0.1–17.2). Compared with the EV‐301 trial [10], there was no significant difference in the frequency of skin reactions (16.6% vs. 14.5%); however, the time to onset was slightly longer with combination therapy (1.61 vs. 0.53 months). These findings highlight the need for close monitoring of skin reactions, particularly in the early stages after EV administration, both in monotherapy and in combination therapy.

**TABLE 1:** Characteristics of patients with SJS/TEN caused by Enfortumab Vedotin.

		Age	Sex	Onset	Treatment	Outcome	Cause of death
2021	Nguyen (8 cases)	71–81	7 M, 1F	Day 9–21 (median11, mean13)	Steroids (7), etanercept (1), IVIG (1)	Expired (2), unknown (6)	Multiple organ failure and septic shock (1), unknown (1)
2022	Singh	47	F	Day 11	Steroids, IVIG, etanercept, diphenhydramine	Alive	—
2022	Birmingham	63	M	Day 22	IVIG	Expired	Multiple organ failure
2023	Mimura	71	M	Day 5	Steroids	Expired	Multiple organ failure
2024	Khanjar	87	M	A few days	Steroids, IVIG, etanercept	Alive	—
2024	Khanjar	81	F	5 weeks	Steroids, IVIG, etanercept	Expired	Overall condition worsening

Abbreviations: IVIG, intravenous immunoglobulin; SJS, Stevens‐Johnson syndrome; TEN, toxic epidermal necrosis.

Severe GI manifestations occur in 10% of SJS/TEN cases [2] and can be fatal. Jha et al. [11] summarized 25 cases of SJS/TEN with GI involvement, reporting symptoms such as GI bleeding, diarrhea, abdominal pain, and vomiting. Mucosal erosions and ulcers primarily affect the colon, followed by the small intestine and stomach. Although the pathogenesis of GI symptoms with TEN remains unclear, mucosal injury caused can occur within the intestine, which may lead to intestinal obstruction in some patients due to edema of the intestinal wall, intussusception, and luminal stenosis. GI symptoms tend to appear several weeks after the onset of cutaneous manifestations (0–7 weeks, typically within 2 weeks) and may persist for months. GI symptoms with TEN have primarily been managed conservatively. In addition to standard treatments for TEN, some patients need surgical resection of the intestine or total parenteral nutrition.

In our case, intestinal mucosal damage associated with TEN was clinically suspected. Initially, given the patient's improving skin lesion and his condition with sepsis and febrile neutropenia, we aimed to reduce the steroid dosage quickly. We considered an adverse event of EV unlikely, given the low incidence of colitis in clinical trials (0.3%–0.7%) [12]. Although ischemic or infectious colitis was differential diagnosis and colonoscopy with biopsy was considered ideal, it was contraindicated due to the patient's unstable hemodynamic status and a high risk of intestinal perforation. A potential alternative might be to maintain or more gradually reduce the steroid dosage during the worsening of GI symptoms. Even if cutaneous symptoms improve, careful monitoring for other systemic complications, including GI manifestations, remains essential.

Because managing TEN is challenging once it develops, it is crucial to frequently monitor for adverse events, including skin symptoms, immediately after EV administration.

## 4 Conclusion

We encountered a case of TEN that developed shortly after administration of EV and was further complicated by severe GI bleeding. Even if the skin rash improves, it is crucial to monitor for other potentially fatal symptoms, such as GI manifestations. Prompt recognition and management of adverse events are essential to improving the prognosis.

## Consent

Written informed consent was obtained from the patient.

## Conflicts of Interest

The authors declare no conflicts of interest.
